# LncRNA FGD5-AS1 Facilitates the Radioresistance of Breast Cancer Cells by Enhancing MACC1 Expression Through Competitively Sponging miR-497-5p

**DOI:** 10.3389/fonc.2021.671853

**Published:** 2021-06-18

**Authors:** Ji Li, Changjiang Lei, Bineng Chen, Qingfang Zhu

**Affiliations:** ^1^Union Hospital, Tongji Medical College, Huazhong University of Science & Technology, Wuhan, China; ^2^Department of General Surgery, The Fifth Hospital of Wuhan, Wuhan, China; ^3^Department of Rehabilitation Medicine, The 910^th^ Hospital of The People's Liberation Army Joint Logistics Support Unit, Quanzhou, China; ^4^Department of Radiology, China Resources & WISCO General Hospital, Wuhan University of Science and Technology, Wuhan, China

**Keywords:** breast cancer, radiation sensitivity, FGD5-AS1, lncRNA, MACC1

## Abstract

**Background:**

LncRNA-FGD5-AS1, as an oncogene, participates in the development and progress of various cancers. However, the exact role and the molecular mechanisms by which FGD5-AS1 regulates radiosensitivity in breast cancer (BC) remains largely unknown.

**Methods:**

We used X-Ray weekly-dose-increase method to establish radiation-resistance cell lines. Bioinformatics tools analyze the expression of FGD5-AS1 in breast cancer tissue and evaluated the relationship between FGD5-AS1 and clinic-pathological features. CCK-8 and colony formation were used to analyze cell proliferation. Western blotting and qPCR were applied to detect protein and gene expression, respectively. RNA interference was used to knock down the endogenous gene expression. Luciferase reporter system and immunoprecipitates were applied to verify the target of FGD5-AS1.

**Result:**

FGD5-AS1 was overexpressed in BC tissues and radiation-resistance cell lines. Higher levels of FGD5-AS1 predicted poorer clinical characteristics and prognosis. Loss-of-function FGD5-AS1 sensitized BC cells to X-ray, meanwhile, the cell gained radiation-resistance when exogenous FGD5-AS1 was expressed. FGD5-AS1 depletion arrested cells at G0/G1 and triggers cell apoptosis. The starBase database (ENCORI), predicted binding site of miR-497-5p in FGD5-AS1 sequence, and luciferase reporter system and immunoprecipitates verified miR-497-5p was the target of FGD5-AS1. Furthermore, MACC1 was predicted and verified as the target of miR-497-5p. Loss-of-function FGD5-AS1 sensitized ionizing radiation was rescued by the up-regulation of MACC1 and the inhibition of miR-497.

**Conclusion:**

FGD5-AS1 displays an oncogene profile in CRC; patients with high expression of FGD5-AS1 should benefit less from radiotherapy and need a more frequent follow-up. Besides, FGD5-AS1 may be a potential therapeutic target for CRC.

## Introduction

Breast cancer (BC) accounts for the most common cancer by mobility and the fifth leading cause of mortality in China (1). Despite all efforts and notable advances for improving CRC treatment including surgery, target therapy, radiotherapy, endocrine therapy, and chemotherapy, the average 5-year survival rates of stage II and stage III patients were 75% and 61%, respectively (2). Radiation therapy is an integral part of the multidisciplinary management of breast cancer (3). One of the main concerns around radiotherapy is how to improve biological effect without compromising organ at risk (4, 5); however, promoting the therapeutic effect o is still a challenge for radiation oncologists.

Long non-coding RNAs (lncRNAs) are non-protein coding transcripts with a length of more than 200 nucleotides. Of note, it is clear that lncRNAs are important regulators of biological processes, including chromosome structure modulation, epigenetic regulation, transcription, mRNA splicing, and translation (2, 6). lncRNAs may carry out both gene inhibition and activation *via* a range of diverse mechanisms to affect the progress of BC such as H19 (7), SRA (8), Zfas1 (9), BCAR (10), and LSINCT5 (11). However, to date, only a few lncRNAs have been characterized in detail for radiotherapy in BC.

FGD5-AS1 has been identified as an oncogene in multiple cancers. Li D et al. reported FGD5-AS1 promoted cell proliferation and metastasis in colorectal cancer and predicted poor survival (12). Similarly, in oral cancer cells, FGD5-AS1 affected cell proliferation, invasion, and migration, and apoptosis as well (13). Furthermore, the tumorigenic potentialities of FGD-AS1 had been revealed in glioblastoma (14), and melanoma (15), as it was related to poor clinic-pathological features and prognosis.

In the current study, we determined the levels of FGD5-AS1 lncRNA in Breast cancer. Furthermore, to understand the possible mechanism of FGD5-AS1 in radiation sensitivity, we established radiation-resistance cell lines. The effects of abnormal expression of FGD5-AS1 on radiation sensitivity were then evaluated by detecting cell viability, apoptosis. Several studies reported that lncRNAs serving as competitive endogenous RNAs (ceRNAs) and sponging miRNAs in many cancers (12–14). For example, FGD5-AS1 lncRNA, as a ceRNA, was proved to target miR-142-3p, miR-153-3p, and miR-129-5p (11).

Metastasis-associated in colon cancer 1 (MACC1), was first identified by Arlt et al. in colon cancer tissues (16). Scholars reported MACC acted as an oncogene involved in cancer progress and metastasis and predicted a poor prognosis in many cancers (17–25). Moreover, many scholars revealed that MACC1 expression has also been associated with treatment response *via* interrupting Notch pathway (26), β-catenin (27, 28), and HGF/cMet pathway (29). These investigations implicate MACC1 as a multifunctional regulator of cancer progression.

The starBase database (ENCORI), predicted the binding site of miR-497-5p in the FGD5-AS1 sequence, we investigated whether FGD5-AS1 regulates radiation-sensitivity *via* sponging miR-497-5p. Moreover, a regulatory pattern between miR-497-5p and MACC1 was detected. We, therefore, believe that our discoveries would supply a theoretical basis for overcoming the radiation-resistance of breast cancer.

## Materials and Methods

### Patients and Sample

A total of 50 BC and paired normal tissues were collected at the China Resources & WISCO General Hospital, Wuhan University of Science and Technology. All samples were confirmed by two pathologists independently. The samples were frozen in liquid nitrogen immediately after surgical resection and then stored at −80°C for later use. Patients who received any neoadjuvant therapies and had a past-tumor-history were excluded from this study. This study was approved by the Ethics Committees of China Resources & WISCO General Hospital, Wuhan University of Science and Technology. The consent was obtained from the study participants before study commencement *via* signing the Informed Consent Form, and this study was strictly conducted in accordance with the Declaration of Helsinki.

### Cell Culture

All the human colorectal cancer cell lines MCF-7 and MDA-MB-231 were cultured in DMEM medium with 10% fetal bovine serum, 100 unit/ml penicillin, and 100 µg/ml streptomycin at 37°C with 5% CO_2_ under sterile conditions.

### Irradiation of Cells and Development of Radio-Resistant Cell Lines

Cells were irradiated using a Faxitron cabinet X-ray system 43855D (Faxitron X-ray Corporation, IL, USA). Radio-resistant cell lines (MCF-7R and MDA-MB-231R) were established on their parental cell lines. Briefly, an initial dose of 2 Gy was followed by weekly incremental doses of 0.5 Gy for 12 weeks for a total radiation dose of 57 Gy. Cells were subsequently maintained with further weekly doses of 5 Gy.

### Cell Transfection

shRNA were synthesized and subcloned into pLKO.1-TRC-puro plasmid. We applied the lipofectamine 2000 system to transfect the plasmids. Briefly, 8×10^5^ cells were seeded into a 6-well plate and cultured for 24 h, then mix the lipofectamine 2000 and plasmid (5 μg) with OPTI-MEM, sequentially, incubated the mixture for 30 min at room temperature, then added the mixture into each well, complemented the OPTI-MEM up to 1 ml and cultured for 6 h. After 6 h, added DMED with 20% FBS into each well and up to 2 ml medium, continued to culture 48 h for mRNA extraction and protein collection.

The shRNA sequences are: FGD5-AS1: 5′-GCA AUG AUG CGC CAC UAG AUU G-3′; miRNA-497: 5′-ACAAACCACAGUGUGCUGCUG-3′; Negative control:5′-CCA UCA GUC CCA AAU CCA-3′. Mimics sequences:miR-497 mimic (5ʹ-CAG CAG CAC ACT GTG GTTGT-3ʹ) and negative control (5ʹ-GTC GTC CTC TGT CAC CAGACT-3ʹ). MACC1 shRNA were purchased from Santa Cruz (Cat#: SC-89466). To overexpressing MACC1, the whole coding sequence of MACC1 was subcloned into the pZSG vector.

### RNA Extraction and Quantitative Real-Time Polymerase Chain Reaction (qRT-PCR) Assays

Total RNA was extracted by using TRIzol reagent (Invitrogen, Carlsbad, CA) according to the manufacturer’s protocol. The first-strand cDNA was synthesized using a reverse transcription kit (Takara, Dalian, China). GAPDH was used as an internal control.

The PCR primers for lncRNA FGD-AS1 and GAPDH were listed the primers: FGD5-AS1 forward, 5′-AGA AGC GGA GGG GTG AAA AT-3′ and reverse, 5′-CCG CCT TAT AGT TGG CCC TC-3′; GAPDH forward, 5′-AAC GGA TTT GGT CGT ATT G-3′ and reverse, 5′-GGA AGA TGG TGA TGG GAT T-3′. miRNA-497: forward, 5′-CAG CCC TGT CCA GTAGC-3′ and reverse, 5′-GCC TGA CTT TAC TGT TGC-3′; MACC1: forward, 5′-TTC TTT TGA TTC CTC CGG TGA-3′ and reverse, 5′-ACT CTG ATG GGC ATG TGC TG-3′. The relative expression of FGD-AS1 was calculated using the 2^-ΔΔCt^ method. All samples were performed in triplicate.

### Cell Proliferation Assay

For cell proliferation assay, HCT116 and SW480 cells were seeded onto 96-well plates (2× 10^3^ cells/well) and cultured for 24, 48, and 72 h. The incubation was conducted at 37°C. After adding 10 μl CCK8 (Dojindo, Japan) to each well and incubating at 37°C for 2 h, the absorbance at 480 nm was measured by the Rayto-6000 system (Rayto, China), and normalized to that of DMEM medium as control.

### Flow Cytometry Analysis

For cell cycle analysis, the cells were harvested after starving for 6 hours and fixed with cold ethanol overnight and then incubated with propidium iodide and RNAse (BD, USA) in the dark for 15 min; for apoptosis analysis, the cells were harvested after starving for 6 h and washed twice with cold PBS, and stained with FITC-conjugated Annexin V for 20 min and propidium iodide for 15 min in the dark.

The stained cells were assessed by flow cytometry (FACS AriaIII, BD, USA) and analyzed by FlowJo vX.0.7 software (30, 31).

### Western Blot Analysis

RIPA lysis buffer (Beyotime Biotechnology) containing protease inhibitors (Roche) was applied to extract the proteins. Quantification of proteins was carried out applying the BCA™ Protein Assay Kit (Pierce, Appleton, WI, USA). After that, proteins (30 μg/sample) were loaded and electrophoresed with 10% sodium dodecyl sulfate-polyacrylamide gel electrophoresis, followed by the transfer to the polyvinylidene difluoride membranes.

The antibodies were prepared in 5% blocking buffer with a dilution of 1:1000, incubated with the membrane at 4°C overnight, washed twice with TBST, and later cultivated with a secondary antibody (1:2000), which was marked by horseradish peroxidase at room temperature for 2 h. Immobilon Western Chemiluminescent HRP Substrate (Millipore) was used to cover the surface of the membrane. Finally, the signals were captured, and the concentration of the bands was quantified using Image Lab™ software (Bio-Rad Laboratories, Hercules, CA, USA).

### Luciferase Reporter Assay

FGD5-AS1-WT, FGD5-AS1-Mut were respectively subcloned into pGL3 vectors (Promega, Madison, WI), constructing plasmids for further transfection with indicated mimics or siRNAs for 48 h. the cells were digested with restriction enzymes, the amplified PCR product was cloned into the polyclonal site of the reconstructed pGL3 expression vector. Finally, the luciferase activity was detected using the Dual-Luciferase^®^ Reporter assay kit (Promega, Madison, Wisconsin, USA).

### Statistical Analysis

All experiments were performed three times. The results are reported as the mean ± SEM. All statistical analyses were performed using GraphPad Prism version 8.0 (GraphPad Software, La Jolla, CA). The association between FGD5-AS1 expression and clinical characteristics was analyzed by the chi-square tests. Survival was compared by Kaplan-Meier analysis. The difference was calculated by the Student’s t-tests, one-way ANOVA analysis or Mann-Whitney U tests. Correlations were evaluated by Pearson correlation coefficient. A p-value of less than 0.05 was considered statistically.

## Results

### FGD5-AS1 Is Highly Expressed in Radioresistant BC Tissues and Cell Lines

**Figure 1:**
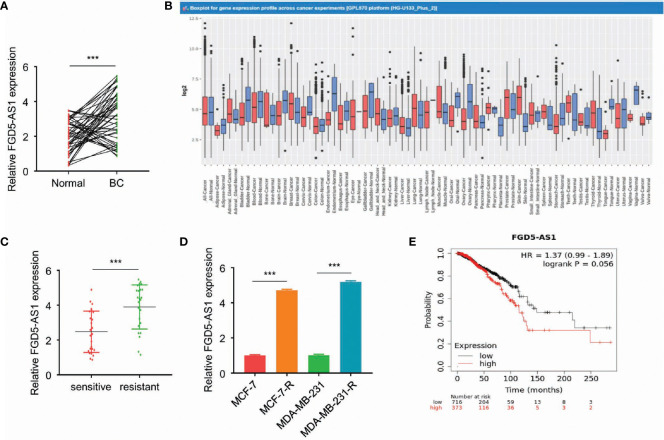
FGD5-AS1 is highly expressed in radioresistant BC tissues and cell lines. **(A)** The expression of FGD5-AS1 in BC tissues (n=50) and corresponding adjacent normal tissues (n=50) *via* qRT-PCR assays. **(B)** GENT2 database showed the significant upregulation of FGD5-AS1 in BC tissues compared with normal tissues (breast-normal) (P < 0.001); **(C)** Examining the expression of FGD5-AS1 in the radio-sensitive group and radio-resistant *via* qRT-PCR assays. As indicated, FGD5-AS1 expression was significantly increased in the radio-resistant group than the radio-sensitive group. **(D)** The expression of FGD5-AS1 in the radio-resistant BC cell lines. As indicated, the expression level of FGD5-AS1 was significantly increased in the radio-resistant BC cells compared to that in their parental cells. **(E)** Using the KM plotter database, to show that higher FGD5-AS1 expression was correlated with worse survival in BC patients. Intra-group comparison ***p < 0.001.

To begin with, we analyzed FGD5-AS1 expression in our patient samples. Quantitative RT-PCR revealed that FGD5-AS1 increased in cancer tissues (**Figure 1A**). Meanwhile, we search the GENT2 database, not to our surprise, FGD5-AS1 expression significantly elevated in BC tissues (P < 0.001, **Figure 1B**). Afterward, we divided the sample into 2 groups as a radiation-sensitive group (n=25) and a radiation-resistant group (n=25) by median OS. Of note, QPCR reveals that FGD5-AS1 expression was remarkably increased in the radiation-resistant group (P < 0.005, **Figure 1C**). As previously described, we successfully established two radiation-resistant BC cell lines MCF-7R and MDA-MB-231R. Continuously, we noted that FGD5-AS1 was up-regulated in the radiation-resistant BC cells (P < 0.005, **Figure 1D**).

The mean value of FGD5-AS1 expression was set as the threshold value, and the BC tissue specimens were divided into the highly-expressed group (n=25) and under-expressed group (n=25). As shown in **Table 1**, higher levels of FGD5-AS1 were positively associated with poor histological grade, advanced tumor stage, lymph node metastasis, and less radiotherapy response. Moreover, the KM plotter database also indicated that higher FGD5-AS1 expression was correlated with worse survival (P < 0.005, **Figure 1E**).

**Table 1:** Correlations of FGD5-AS1 expression with clinicopathologic features of breast cancer.

Factor	FGD5-AS1 expression		*P* value
	low (n=25)	High (n=25)	
Age			0.255
≤50	16	12	
>50	9	13	
Tumor Size			0.087
≤2cm	17	11	
>2cm	8	14	
Tumor Differentiation			0.041
I	19	12	
II	6	13	
Clinical Stage			0.031
I/II	21	14	
III/IV	4	11	
Lymph node metastasis			0.042
Yes	24	19	
No	1	6	
Tumor response			0.036
CR	0	0	
PR	3	1	
SD	12	5	
PD	10	19	

### FGD5-AS1 Strengthens the Radio-Resistance of BC Cell

**Figure 2:**
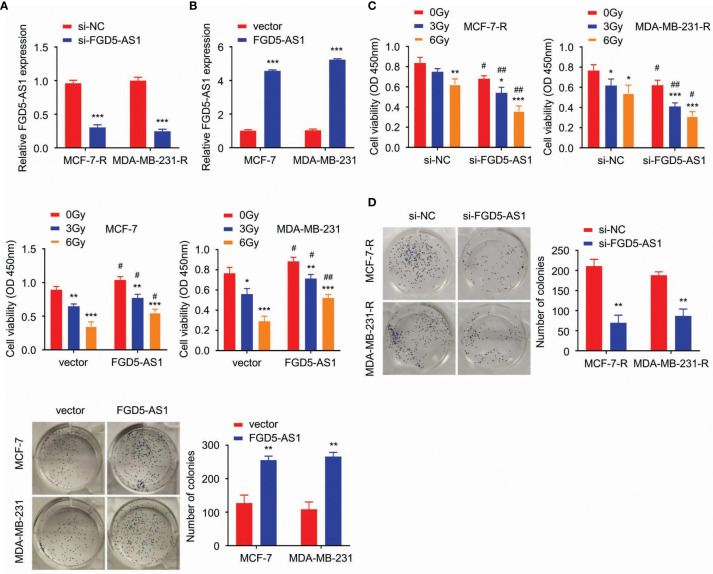
FGD5-AS1 strengthens the radio-resistance of BC cells. **(A)** MCF-7-R and MDA-MB-231-R were transfected with specific siRNA of human FGD5-AS1. qRT-PCR to confirm the downregulation of FGD5-AS1 in transfected BC cells. **(B)** As indicated, basing on qPCR, FGD5-AS1 exogenous expression was carried out in MCF-7 and MDA-MB-231 cells. **(C)** cell viability was detected by CCK-8. Cells were exposed to different doses of ionizing radiation (0, 3, and 6 Gy). FGD5-AS1 deficiency significantly promoted radiation sensitivity of MCF-7-R or MDA-MB-231-R cells; while FGD5-AS1 exogenous expression attenuated the radiation sensitivity of MCF-7 or MDA-MB-231 cells. **(D)** Colony formation assays, BC cells treated with 2 Gy ionizing radiation. Colony formation assays displayed that FGD5-AS1 deficiency significantly promoted radiation sensitivity of MCF-7-R or MDA-MB-231-R cells; while FGD5-AS1 exogenous expression attenuated the radiation sensitivity of MCF-7 or MDA-MB-231 cells. Intra-group comparison *p < 0.05, **p < 0.01, ***p < 0.001; Inter-group comparison ^#^p < 0.05, ^##^p < 0.01.

We successfully transfected FGD5-AS1 siRNA and expression plasmids into MCF-7-R or MDA-MB-231-R cells (P < 0.005, **Figures 2A, B**). The cells were exposed to different doses of ionizing radiation (0, 3, and 6 Gy), and confirmed that the radiation was conducted as a dose-dependent suppressive effect. CCK-8 and colony formation assays results indicated the deficiency of FGD5-AS1 facilitated cellular radiation-sensitivity and could be rescued by exogenous FGD5-AS1 expression (P < 0.005, **Figures 2C, D**).

### FGD5-AS1 Depletion Triggers Cell Apoptosis in BC Cells

**Figure 3:**
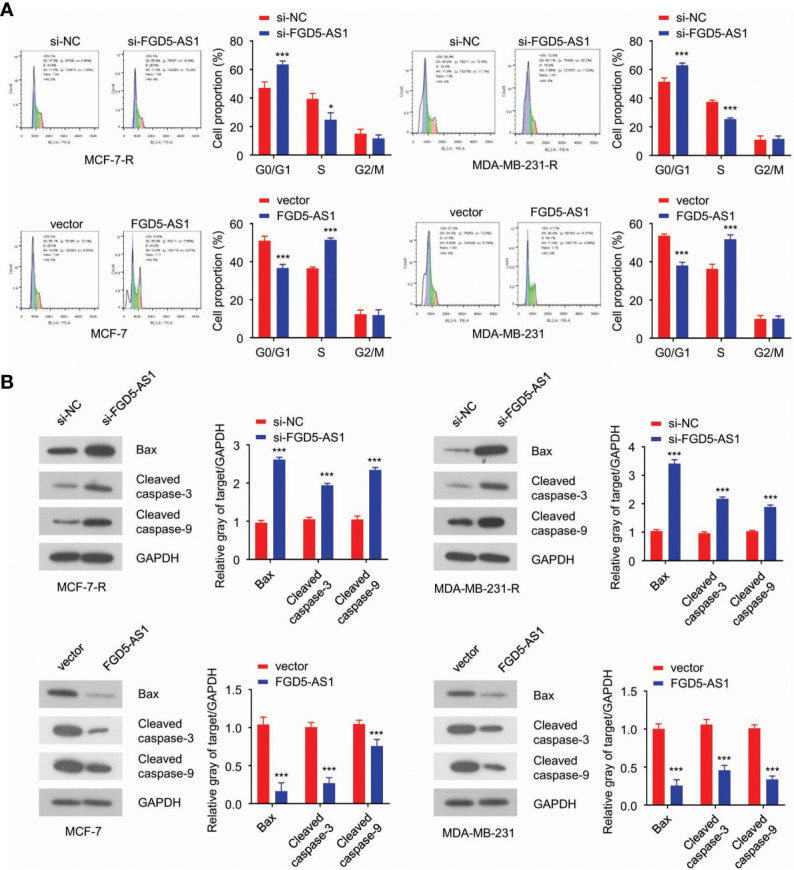
FGD5-AS1 depletion triggers cell apoptosis in BC cells. **(A)** flow cytometry analysis indicated downregulation of FGD5-AS1 arrested MCF-7-R or MDA-MB-231-R cells at G0/G1and induced much more apoptosis after treated with 2 Gy of ionizing radiation. Meanwhile, overexpression of FGD5-AS1 arrested MCF-7 and MDA-MB-231 at the S phase, and less apoptosis was observed after ionizing radiation. **(B)** WB showed that knockdown of FGD5-AS1 significantly increased the protein levels of Bax, cleaved caspase-3, and caspase-9 in MCF-7-R or MDA-MB-231-R cells treated with 2 Gy of ionizing radiation. Overexpression of FGD5-AS1 significantly decreased the protein levels of Bax, cleaved caspase-3, and caspase-9 in MCF-7-R or MDA-MB-231-R cells treated ionizing radiation. Intra-group comparison *p < 0.05, ***p < 0.001.

Next, we wondered how FGD5-AS1 performed its biological function in radiation-resistant cells. FACS results revealed that loss-of-function FGD5-AS1 arrested MCF-7-R or MDA-MB-231-R at the G0/G1 phase (**Figure 3A**) and induced more apoptosis when exposed to 2 Gy X-ray (**Figure 3B**).

### FGD5-AS1 Directly Binds to miR-497-5p and Suppresses Its Expression in BC Cells

**Figure 4:**
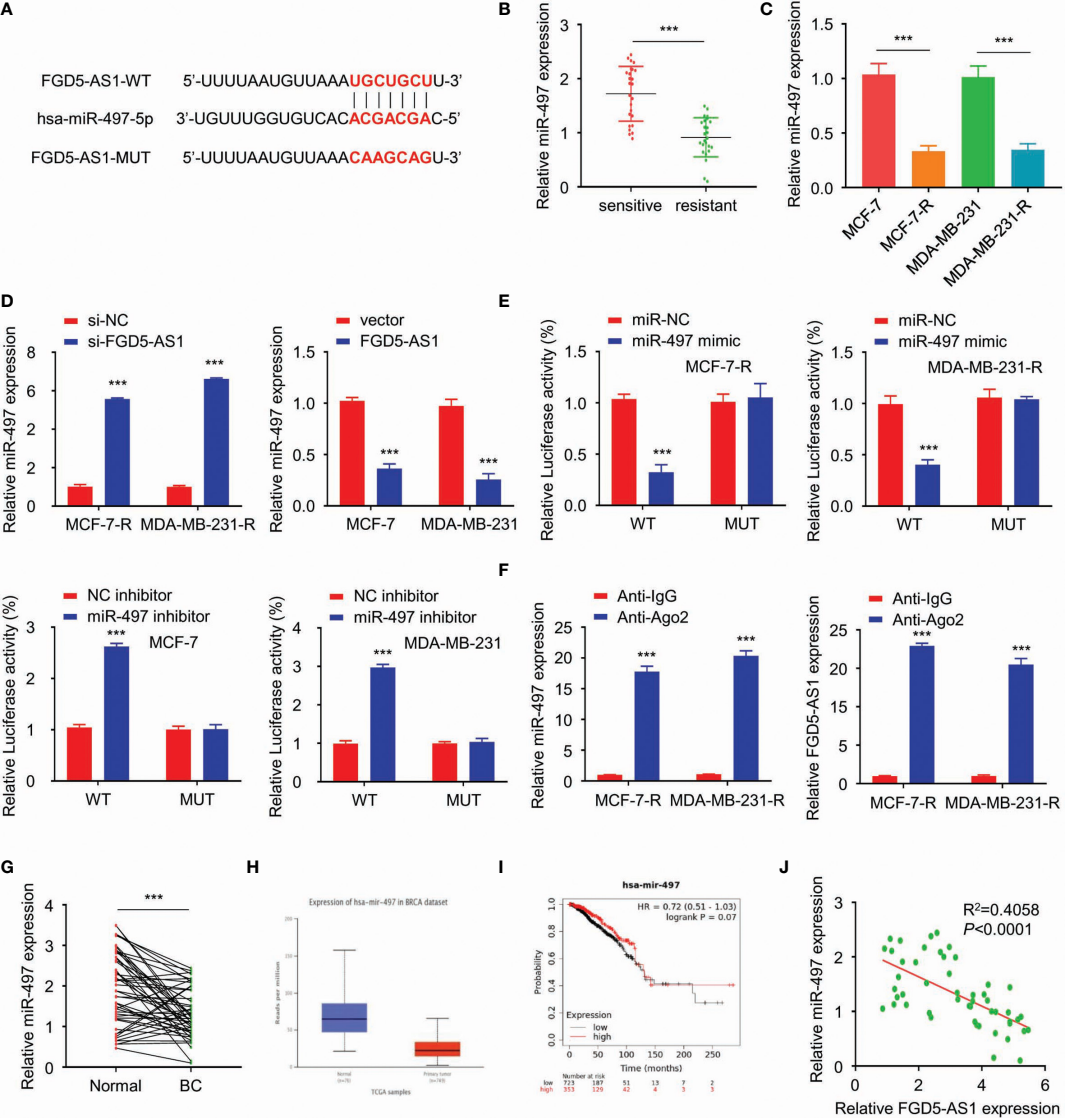
FGD5-AS1 directly binds to miR-497-5p and suppresses its expression in BC cells. **(A)** Using the starBase database to predict the binding site of miR-497-5p in the FGD5-AS1 sequence. **(B)** qRT-PCR assays showed that miR-497 expression was significantly decreased in the radio-resistant BC tissues group (n=25). **(C)** qRT-PCR assays indicated the lower levels of miR-497 in MCF-7-R or MDA-MB-231-R cells than MCF-7 or MDA-MB-231 cells. **(D)** Using qRT-PCR assays revealed that FGD5-AS1 exogenous expression significantly reduced the expression of miR-497. Meanwhile, FGD5-AS1 silencing significantly upregulated the expression of miR-497. **(E)** Using the luciferase reporter assays, miR-497 mimic significantly reduced the luciferase activity of the pGL3-FGD5-AS1-WT and FGD5-AS1-MT cell were had not been affected. In contrast, miR-497 Inhibitor significantly promoted the luciferase activity of the pGL3-FGD5-AS1-WT, as the same, FGD5-AS1-MT cell were had not been affected. **(F)** RNA binding protein immunoprecipitation against Ago2 indicated that FGD5-AS1 or miR-497 were significantly enriched in Ago2 pellets. **(G)** qRT-PCR assays showed that miR-497 levels were significantly downregulated in BC tissues. **(H)** UALCAN database indicated the significant downregulation of miR-497 in TCGA BC tissues (P<1E-12). **(I)** KM plotter showed that lower miR-497 expression was significantly correlated with worse survival in BC patients. **(J)** PCR showed a negative relationship between FGD5-AS1 and miR-497 expression levels in BC tissues (n=50). Intra-group comparison ***p < 0.001.

Afterward, we analyzed the targets of FGD-AS1 *via* starBase2. Among the targets discovered, miR-497-5p got a high score and the predicted binding site of FGD5-AS1 and miR-497-5p was listed in **Figure 4A**. QPCR revealed that miR-497-5p expression was significantly decreased in the radiation-resistant BC tissues and cells (P < 0.005, **Figures 4B, C**). Also, miR-497-5p was dramatically attenuated in FGD-AS1 deficient cells and rescued by exogenous FGD5-AS1 gene transfected (**Figure 4D**).

We used a pGL3-FGD5-AS1-WT and pGL3-FGD5-AS1-Mut based double luciferase reporter to figure out the target of FGD5-AS1. As a result, the luciferase activity of the pGL3-FGD5-AS1-WT was significantly attenuated when cotransfected with miR-497 mimic. Then, the miR-497 mimic was failed to affect the luciferase activity of the pGL3-FGD5-AS1-MT. Moreover, the luciferase activity of the pGL3-FGD5-AS1-WT was significantly promoted when miR-497 cotransfected, while, miR-497 was unable to affect the luciferase activity of the pGL3-FGD5-AS1-MT (**Figure 4E**).

RNA binding protein immunoprecipitation experiments were performed on MCF-7-R or MDA-MB-231-R cells extracts by the Ago2 antibody. Not to our surprise, FGD5-AS1 or miR-497 were significantly enriched in Ago2 pellets relative to control IgG immunoprecipitates (**Figure 4F**). We investigated that miR-497 levels were significantly downregulated in BC tissues (**Figures 4G, H**) and lower expression of miR-497 predicted an unfavored prognosis (**Figure 4I**). Also, our samples showed a negative relationship between FGD5-AS1 expression levels and miR-497 expression (**Figure 4J**). Taken together, FGD5-AS1 may act as an endogenous sponge for miR-497 to suppress its expression in BC cells.

### FGD5-AS1 Competitively Sponges miR-497 to Enhance MACC1 Expression in BC Cells

**Figure 5:**
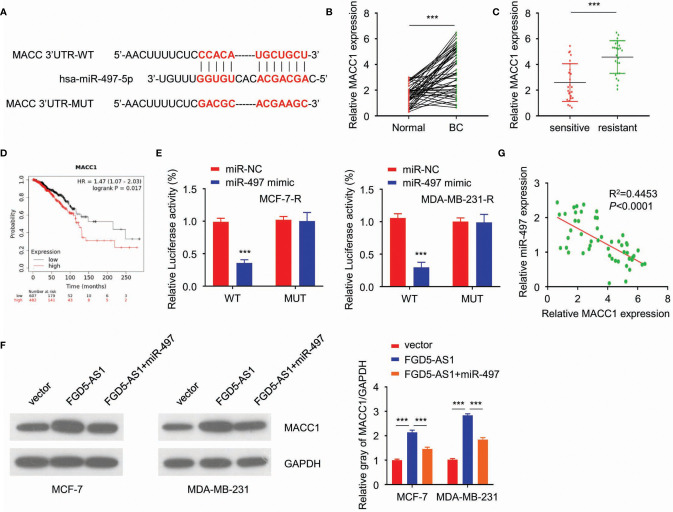
FGD5-AS1 competitively sponges miR-497 to enhance MACC1 expression in BC cells. **(A)** TargetScan predicted the potential binding between miR-497 and MACC1 sequence. **(B)** qRT-PCR assays indicated that MACC1 levels were significantly upregulated in BC tissues (n=50). **(C)** qRT-PCR assays showed that MACC1 expression was significantly increased in the radio-resistant BC tissues group (n=25). **(D)** KM plotter showed that higher MACC1 expression was significantly correlated with worse survival in BC patients. **(E)** Luciferase reporter assays indicated the direct binding between miR-497 and MACC1 in MCF-7-R and MDA-MB-231-R cells. **(F)** WB showed FGD5-AS1 exogenous expression increased MACC1 protein expression and could be reduced by the overexpression of miR-497. **(G)** qRT-PCR assays indicated the negative relationship between miR-497 expression and MACC1 mRNA expression levels in BC tissues (n=50). Intra-group comparison ***p < 0.001.

Continuously, we analyzed the targets of miR-497 *via* starBase2. Among the targets discovered, MACC1 got a high score, and the predicted binding site of miR-497-5p and MACC1 was listed in **Figure 5A**. Besides, studies have shown that the up-regulation of MACC1 promotes tolerance to radiotherapy. Therefore, this study investigated whether FGD5-AS1 affects the protein stability of MACC1 by regulating the expression of miR-497 in BC cells. To begin with, we found that MACC1 was elevated in BC tissues and radiation-resistant cells (**Figures 5B, C**) and predicted a poorer prognosis (**Figure 5D**). Also, luciferase reporter assays indicated the direct binding between miR-497 and MACC1 in MCF-7-R or MDA-MB-231-R cells (**Figure 5E**). Of note, MACC1 was elevated by exogenous FGD5-AS1 expression and rescued by miR-497 inhibition (**Figure 5F**). Likewise, we observed a negative relationship between miR-497 and MACC1 expression levels in BC tissues. These findings demonstrated that FGD5-AS1 could induce the expression of MACC1 *via* acting as a ceRNA to sponge miR-497 in BC cells (**Figure 5G**).

### FGD5-AS1 Induces MACC1 Expression to Promote the Radio-Resistance of BC Cells Through Competitively Sponging miR-497

**Figure 6:**
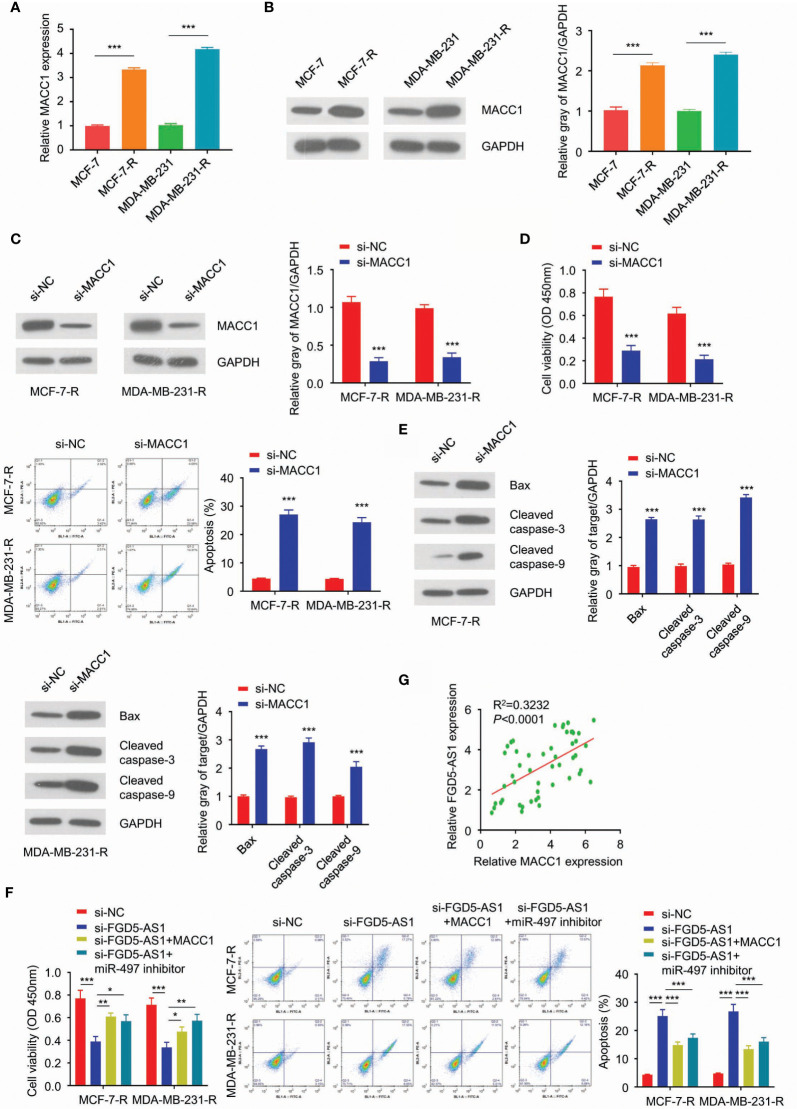
FGD5-AS1 induces MACC1 expression to promote the radio-resistance of BC cells through competitively sponging miR-497. **(A)** qRT-PCR assays showed that mRNA levels of MACC1 were significantly upregulated in MCF-7-R and MDA-MB-231-R cells. **(B)** WB analysis showed that the protein level of MACC1 was increased in the radio-resistant BC cells. **(C)** Transfect MCF-7-R or MDA-MB-231-R cells with human MACC1 siRNA and WB analysis showed the downregulation of MACC1 in transfected cells. **(D)** CCK-8 assay, colony formation assay, and flow cytometry analysis indicated that MACC1deficiency decreased the survival fraction with ionizing radiation, and led to more apoptosis in BC cells. **(E)** The western blotting analysis showed MACC1 deficiency significantly increased the protein levels of Bax, cleaved caspase-3, and caspase-9 in MCF-7-R or MDA-MB-231-R cells. **(F)** CCK-8 assays, clone formation assays, and flow cytometry analysis, to show that knockdown of FGD5-AS1 decreased the survival fraction and induced more apoptosis in BC cells. However, these phenotypes were significantly rescued by MACC1 exogenous expression and the inhibition of miR-497. **(G)** Spearman’s correlation analysis showed the positive correlation between FGD5-AS1 and MACC1 expression in 50 BC tissues, as measured by qRT-PCR assays. Intra-group comparison *p < 0.05, **p < 0.01, ***p < 0.001.

Further experiments exhibited MACC1 was significantly upregulated in MCF-7-R and MDA-MB-231-R cells (**Figures 6A–C**). After we interrupting the function of MACC1, and MCF-7-R and MDA-MB-231-R cells triggered the apoptosis pathway and regained sensitivity to the ionizing radiation (**Figures 6D, E**). As previously described, FGD5-AS1 deficiency was able to sensitize the cells to radiation. This phenotype could be rescued by exogenous MACC1 expression or miR-497 depletion (**Figure 6F**). Similarly, we found a positive relationship between miR-497 and MACC1 expression levels in BC tissues. To sum up, lncRNA FGD5-AS1 promoted the radioresistance of BC cells by upregulating MACC1 expression through competitively sponging miR-497.

## Discussion

Recent studies revealed that lncRNAs acted as a key regulator in the pathogenesis, progression, and phenotype development of breast cancer (32). Growing data delineated LncRNAs dysregulated in breast cancer (33–37). As previously described, lncRNA FGD5-AS1 participated as an essential mediator in various cancer. As we reviewed the published paper, lncRNA FGD5-AS1 might serve as a prognosis factor of colon cancer, oral cancer, and melanoma (38–43). Scholars araised interest of lncRNA FGD5-AS1 from 2018, and reported different molecular mechanism of lncRNA FGD5-AS1 in various cancer, including: regulating miR-140-5p/WEE1 axis (44), miR-153-3p/CITED2 (40), ERK/AKT signaling (45), MCL1 (42), Wnt/β-catenin (28, 46), miR-103a-3p/TPD52 (47). However, the function of lncRNA FGD5-AS1 regarding breast cancer remained rarely studied.

More than 50% of breast cancer patients would undergo radiotherapy during their course of treatment. Radiotherapy, in most cases, through X-ray ionizing radiation, inducing damage of DNA *via* directly or indirectly attacking the backbone of DNA and triggering a sequence of cellular response (12, 48, 49).

**Figure 7:**
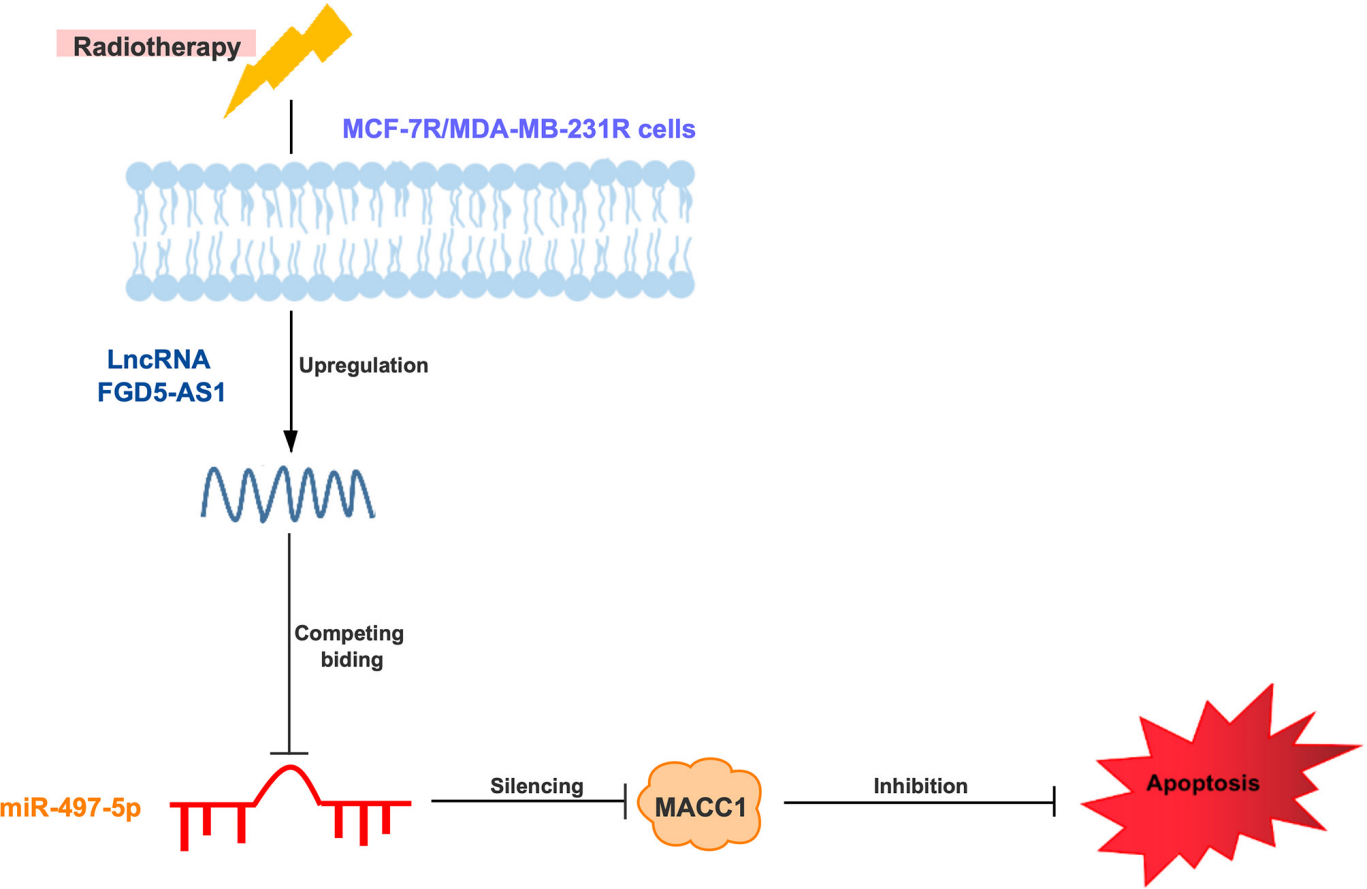
Schematic diagram of lncRNA FGD5-AS1/miR-497-5p/MACC1 axis to regulate MCF-7 or MDA-MB-231 cells radioresistance.

As showing in the schematic diagram (**Figure 7**), we found FGD5-AS1 over-expression reduced cell apoptosis and induced radioresistance in BC cells. miR-497-5p was identified as a potential target of FGD-AS1. Among the targets discovered, got a high score and the predicted binding site of FGD5-AS1 and miR-497-5p. Further, our data indicated that FGD5-AS1 competitively sponges miR-497 to enhance MACC1 expression in BC cells. After we interrupting the function of MACC1, and breast cancer cells triggered the apoptosis pathway and regained sensitivity to ionizing radiation. This phenotype could be rescued by exogenous MACC1 expression or miR-497 depletion. Similarly, we found a positive relationship between miR-497 and MACC1 expression levels in breast tissues.

MACC1 has been well known as a key oncogenesis regulator and biomarker of tumor metastasis in a broad panel of cancer entities, associated with poor prognosis (29, 50–54). In 2013, Huang et al. reported serum MACC1 presented a diagnostic and prognostic value in breast cancer (55); Aiko Sueta et al. revealed MACC1 was failed to affect the MET pathway in the breast (56), which differed from colon cancer. Up to 2020, there was 9 articles showed MACC1 might serve as a good biomarker for breast cancer, and our result keeps consistency with previously reported (25, 55–59).

Additionally, we found that MACC1 promoted cell progress and radiosensitivity through antagonizing miR-497. FGD5-AS1 deficiency was able to sensitize the cells to radiation. This phenotype could be rescued by exogenous MACC1 expression or miR-497 depletion, which robustly suggested that they are connected in a loop. Besides, previous studies have reported that MACC1 exerts an inhibitory effect on drug resistance (60). As reported, FGD5-AS1 deficiency can promote chemoresistance *via* triggering the PI3K pathway, Wnt/β-catenin et al. (41, 42, 45).

The radiation-induced apoptotic mitochondria pathway is regulated by the Bcl-2 family. The Bcl-2 family may act as pro-apoptotic. Bax expression triggers the mitochondrial outer membrane permeabilization and releases cytochrome C to the cytoplasm. As a consequence, activation of the procaspase 9 through with apoptosome leads to cell death (61–63). In our data, we observed that LncRNA FGD5-AS1 deficiency sensitized the cell to X-ray *via* activating the BAX, Caspase 3, and Caspase 9, which indicate cell apoptosis pathway blockade is the main mechanism of LncRNA FGD5-AS1 induced radioresistance.

However, our study have some limitations. Currently, the expression of FGD5-AS1 was detected in limited number of tissue samples due to the number of available patient participants. Increased number of respondents shall be recruited in future studies. Secondly, the downstream effectors that are essential to FGD5-AS1-regulated radiation-resistance of breast cancer cells need to be thoroughly explored. Finally, we also need to further elucidate the regulatory role of MACC1 in radiation-resistance breast cancer.

In conclusion, the present results suggested that the FGD5-AS1/miR-497/MACC1 axis contributed to radiation resistance in breast cancer. These findings indicated patients with high expression of LncRNA FGD5-AS1 might gain more benefit from radiotherapy.

## Data Availability Statement

The original contributions presented in the study are included in the article/supplementary materials, further inquiries can be directed to the corresponding author/s.

## Ethics Statement

This study was approved by the Ethics Committees of China Resources & WISCO General Hospital, Wuhan University of Science and Technology. The consent was obtained from the study participants before study commencement *via* signing the Informed Consent Form, and this study was strictly conducted in accordance with the Declaration of Helsinki. The patients/participants provided their written informed consent to participate in this study.

## Author Contributions

QZ mainly participated in literature search, study design, writing. JL mainly participated in data collection, data analysis and data interpretation. CL conducted the most of cell experiment. BC maily participated in the critical revision. All authors contributed to the article and approved the submitted version. The authors declare that they don't have any conflict and interests in the study.

## Conflict of Interest

The authors declare that the research was conducted in the absence of any commercial or financial relationships that could be construed as a potential conflict of interest.
