# Corrigendum: LncRNA FGD5-AS1 Facilitates the Radioresistance of Breast Cancer Cells by Enhancing MACC1 Expression Through Competitively Sponging miR-497-5p

**DOI:** 10.3389/fonc.2021.750074

**Published:** 2021-08-24

**Authors:** Ji Li, Changjiang Lei, Bineng Chen, Qingfang Zhu

**Affiliations:** ^1^Union Hospital, Tongji Medical College, Huazhong University of Science & Technology, Wuhan, China; ^2^Department of General Surgery, The Fifth Hospital of Wuhan, Wuhan, China; ^3^Department of Rehabilitation Medicine, The 910^th^ Hospital of The People’s Liberation Army Joint Logistics Support Unit, Quanzhou, China; ^4^Department of Radiology, China Resources & WISCO General Hospital, Wuhan University of Science and Technology, Wuhan, China

**Keywords:** breast cancer, radiation sensitivity, FGD5-AS1, lncRNA, MACC1

In the original article, we neglected to mark Changjiang Lei and Bineng Chen as the co-first authors. The co-first authorship indication has now been added to Changjiang Lei and Bineng Chen.

The authors apologize for this negligence and state that this does not change the scientific conclusions of the article in any way. The original article has been updated.

## Publisher’s Note

All claims expressed in this article are solely those of the authors and do not necessarily represent those of their affiliated organizations, or those of the publisher, the editors and the reviewers. Any product that may be evaluated in this article, or claim that may be made by its manufacturer, is not guaranteed or endorsed by the publisher.

